# Opposite Contractile Effects of Amphetamine-Related Hallucinogenic Drugs in the Isolated Human Atrium

**DOI:** 10.3390/ijms25168887

**Published:** 2024-08-15

**Authors:** Joachim Neumann, Britt Hofmann, Ulrich Gergs

**Affiliations:** 1 Institute for Pharmacology and Toxicology, Medical Faculty, Martin Luther University Halle-Wittenberg, 06097 Halle, Germany; ulrich.gergs@medizin.uni-halle.de; 2 Department of Cardiac Surgery, Mid-German Heart Center, University Hospital Halle, 06097 Halle, Germany; britt.hofmann@uk-halle.de

**Keywords:** DOM, DOI, mephedrone, human atrium

## Abstract

The present study examined three hallucinogenic amphetamine derivatives, namely, 2,5-dimethoxy-4-iodoamphetamine (DOI) as well as 2,5-dimethoxy-4-methylamphetamine (DOM) and 4-methylmethcathinone (mephedrone). The objective of this study was to test the hypothesis that DOI, DOM, and mephedrone would increase the contractile force in isolated human atrial preparations in a manner similar to amphetamine. To this end, we measured contractile force under isometric conditions in electrically stimulated (1 Hz) human atrial preparations obtained during open surgery. DOI and DOM alone or in the presence of isoprenaline reduced the contractile force concentration-dependently in human atrial preparations. These negative inotropic effects of DOM and DOI were not attenuated by 10 µM atropine. However, mephedrone increased the contractile force in human atrial preparations in a concentration- and time-dependent manner. Furthermore, these effects were attenuated by the subsequent addition of 10 µM propranolol or pretreatment with 10 µM cocaine in the organ bath. Therefore, it can be concluded that amphetamine derivatives may exert opposing effects on cardiac contractile force. The precise mechanism by which DOI and DOM exert their negative inotropic effects remains unknown at present. The cardiac effects of mephedrone are probably due to the release of cardiac noradrenaline.

## 1. Introduction

**Figure 1:**
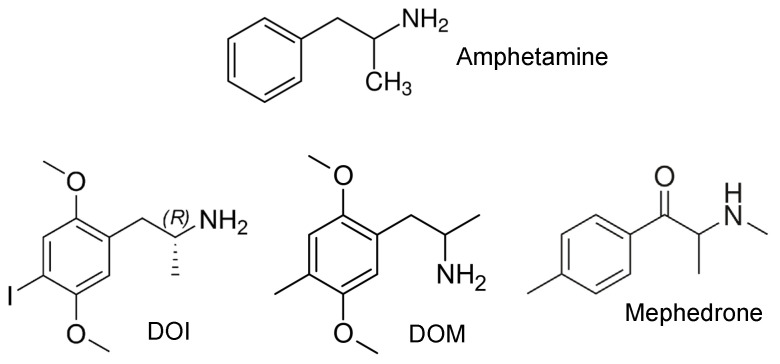
Structural formulae of amphetamine, DOI, DOM, and mephedrone.

Amphetamine has been demonstrated to enhance contractile force in isolated cardiac preparations from mice and humans through the release of endogenous noradrenaline from the heart [1]. In basic neuropharmacological research, amphetamine derivatives (Figure 1) such as 2,5-dimethoxy-4-iodoamphetamine (DOI) and 2,5-dimethoxy-4-methylamphetamine (DOM) are employed as model compounds to investigate the hallucinogenic effects.

For example, for DOI (Figure 1), Sadzot et al. described potent hallucinogenic effects in humans [2]. Subsequently, it was proposed that the mechanism underlying the hallucinogenic effects is mediated via the 5-HT_2A_ serotonin receptor [3]. Further biochemical studies demonstrated that DOI exhibits a high affinity for a number of additional receptors, including β_1_-adrenoceptors (Ki = 591 nM), β_2_-adrenoceptors (Ki = 140 nM), α_2A_-adrenoceptors (Ki = 74 nM), α_2B_-adrenoceptors (Ki = 340 nM), 5-HT_2A_ serotonin receptors (Ki = 165 nM), 5-HT_2B_ serotonin receptors (Ki = 336 nM), and 5-HT_2C_ serotonin receptors (Ki = 46 nM) [4]. Of the receptors listed above, only stimulation by β_1_- and β_2_-adrenoceptors has been demonstrated to have a positive inotropic effect in the human heart. The literature therefore suggests that DOI may increase the contractile force in the human atrium through the release of noradrenaline and the subsequent stimulation of β-adrenoceptors. However, speculatively, it may also lead to serotonin release and subsequent activation of 5-HT_4_ serotonin receptors.

DOM (Figure 1) is closely related to DOI in terms of its chemical structure and is also known to induce potent hallucinogenic effects in humans [5,6]. The affinity profile of DOM was similar to that of DOI, for example, with a Ki of 507 nM for 5-HT_2A_ serotonin receptors or with a Ki of 48.9 nM for β_2_-adrenoceptors. However, no affinity was observed for β_1_-adrenoceptors (Ki > 10,000 nM) [4]. Others found that DOM is a partial agonist at 5-HT_2A_ serotonin receptors in the brain [7]. Furthermore, DOM reduced blood pressure and heart rate, at least in rats and cats, and these effects were suggested to be mediated via the central nervous system [8,9].

Mephedrone (Figure 1) is a cathinone derivative within the amphetamines class of compounds, differing from both DOI and DOM in its chemical structure. Mephedrone has a high affinity for 5-HT_2A_, 5-HT_2B_, and 5-HT_2C_ serotonin receptors [10], but there is no binding to adrenergic or muscarinic receptors [10]. In contrast, mephedrone has been demonstrated to inhibit almost all important monoamine transporters such as serotonin, dopamine, and noradrenaline transporters as well as vesicular monoamine transporters [11,12,13].

In light of these findings, we hypothesized that DOI, DOM, and mephedrone may influence the contractile force in the human atrium either by directly binding to β-adrenergic receptors or by releasing noradrenaline and subsequently increasing the contractile force in the human atrium, as previously shown for amphetamine [1].

## 2. Results

### 2.1. Effects of DOI, DOM, and Mephedrone Alone

**Figure 2:**
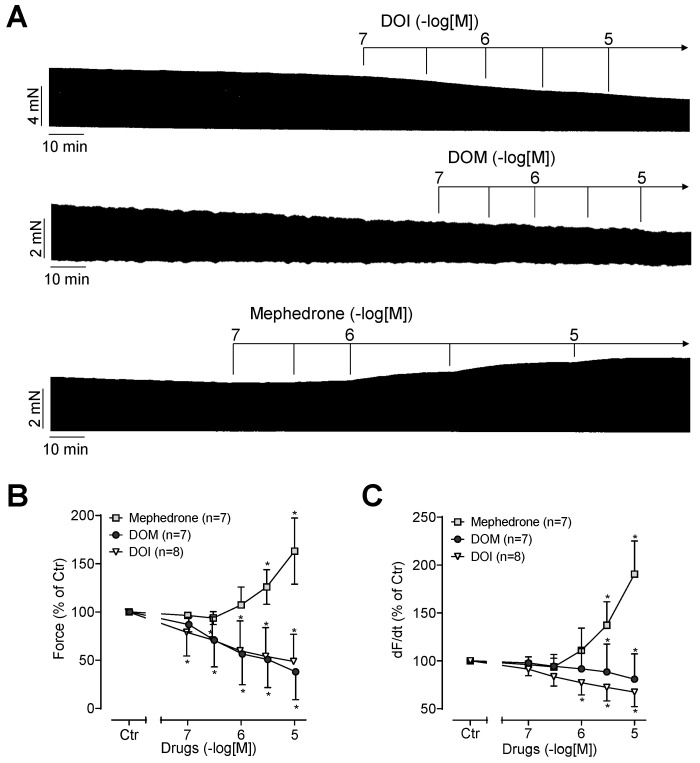
(**A**) Original recordings of the inotropic effects of DOI, DOM, and mephedrone in millinewton (mN) in electrically stimulated human right-atrial preparations. Horizontal bars indicate time axis in minutes (min). (**B**) Force of contraction in percent of control (Ctr; pre-drug value). (**C**) Maximum rate of contraction (dF/dt) in percent of control. * *p* < 0.05 versus Ctr. Data are shown as mean ± SD, and expressed as the percentage of control. The numbers in brackets indicate the number of experiments.

**Figure 3:**
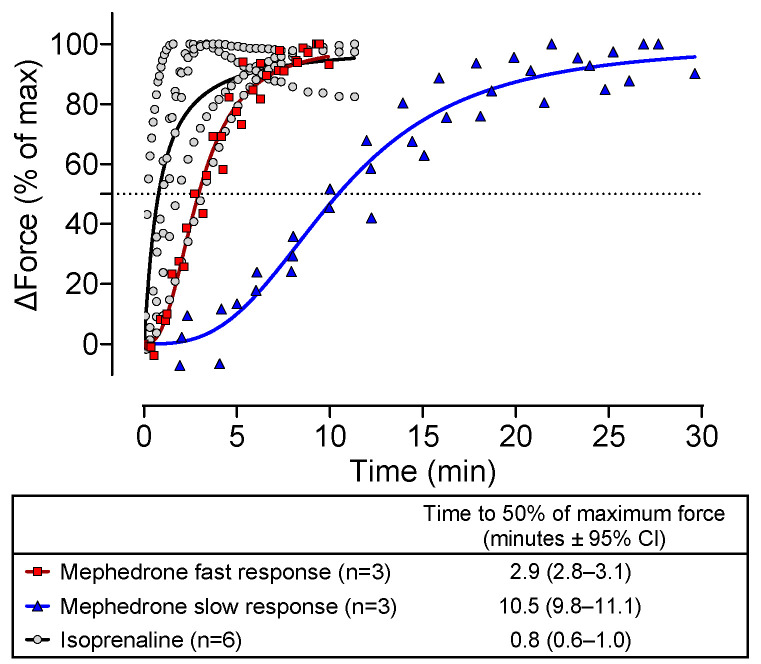
Time-dependent increase in contractile force for mephedrone (10 µM) and isoprenaline (0.1 µM). Force is expressed as delta force as percentage of maximum tension. The table below the graph contains the figure legends along with the time at which 50% of tension is reached together with the 95% confidence interval (CI). The numbers in brackets indicate the number of experiments.

DOI or DOM alone reduced the contractile force in a concentration- and time-dependent manner in the human atrial preparations. This is seen in the original recordings in Figure 2A and summarized in Figure 2B. Accordingly, DOI and DOM also reduced the rate of tension development under these conditions, as demonstrated by the statistical analysis presented in Figure 2C. In contrast, mephedrone alone increased the contractile force in a concentration- and time-dependent manner as well as the rate of tension development (Figure 2). This is evident in the original recording depicted in Figure 2A and in the summarized data presented in Figure 2B,C. Figure 3 illustrates in typical original recordings the time dependence of the developed force for mephedrone and, for comparison, for isoprenaline, which directly activates the β-adrenoceptor. The time of application of the drugs was set to zero, and the time until the maximum tension was reached for a certain concentration was measured. It is noteworthy that the preparations could be divided into slow and fast responders, since the release of noradrenaline was probably different in the atria from different patients for unknown reasons (Figure 3).

### 2.2. Effects of DOI, DOM, and Mephedrone after β-Adrenergic Prestimulation

**Figure 4:**
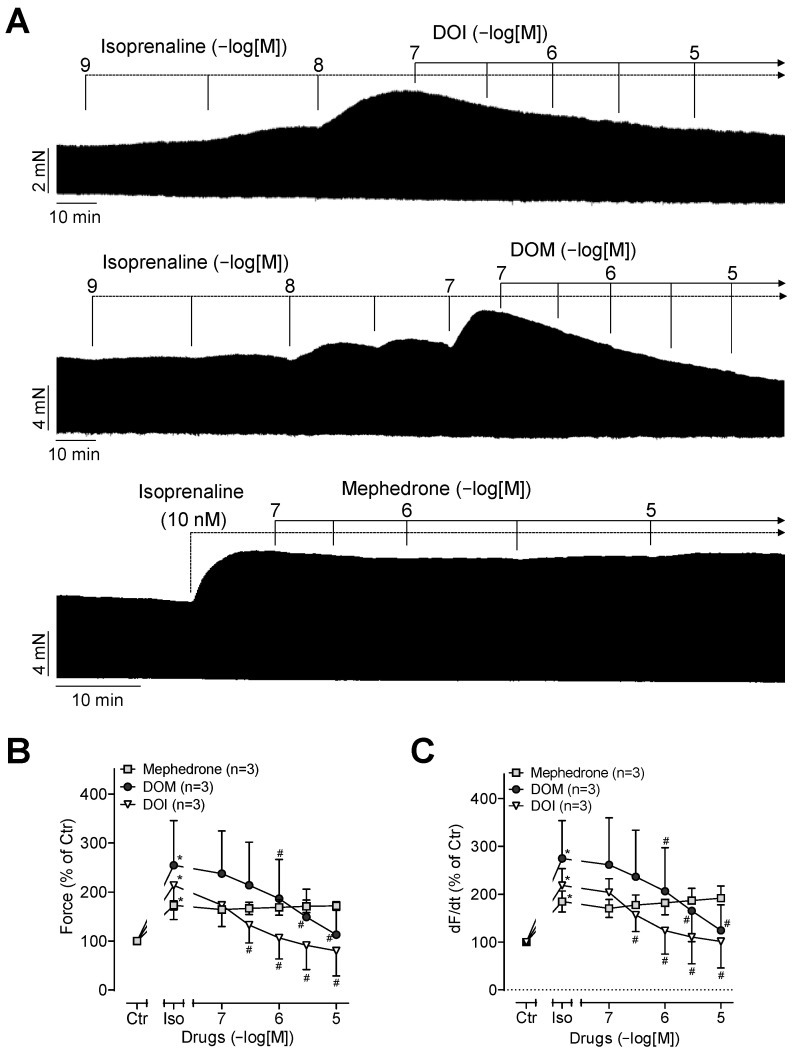
(**A**) Original recordings of the inotropic effects of DOI, DOM, and mephedrone after pre-stimulation with isoprenaline in millinewton (mN) in electrically stimulated human right-atrial preparations. Horizontal bars indicate time axis in minutes (min). (**B**) Force of contraction in percent of control (Ctr; pre-drug value). (**C**) Maximum rate of contraction (dF/dt) in percent of control. * *p* < 0.05 versus Ctr; # *p* < 0.05 versus isoprenaline (Iso). Data are shown as mean ± SD, and expressed as the percentage of control. The numbers in brackets indicate the number of experiments.

It was previously demonstrated that drugs such as lysergic acid diethylamide (LSD) exert an anti-β-adrenergic effect [14]. These observations prompted us to first stimulate the contractile force with the β-adrenoceptor agonist isoprenaline and subsequently add DOI, DOM, or mephedrone. This is shown in the original recordings presented in Figure 4A. It was observed that while isoprenaline increased the contractile force (Figure 4A), additional DOI or DOM did not augment this force but rather diminished it (Figure 4A). However, mephedrone had no additional inotropic effect under these conditions (Figure 4A). The results of several such experiments are summarized in Figure 4B with respect to contractile force. Consequently, isoprenaline also increased the rate of tension development (Figure 4C). Additionally applied DOI or DOM reduced the rate of tension development as did the contractile force, and again, mephedrone did not further alter this parameter (Figure 4C).

### 2.3. Mechanisms of DOI, DOM, and Mephedrone

**Figure 5:**
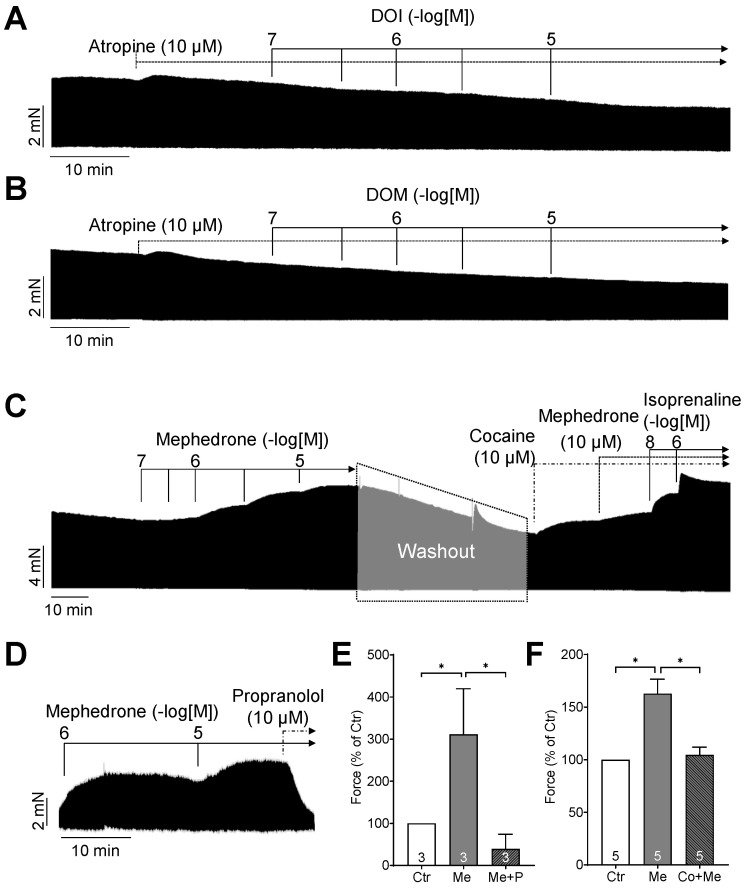
Original recordings of the inotropic effects of DOI (**A**) and DOM (**B**) after pre-incubation with 10 µM atropine in millinewton (mN) in electrically stimulated human right-atrial preparations. (**C**) Original recording of the positive inotropic effect of mephedrone alone, and mephedrone in the additional presence of 10 µM cocaine in mN. Finally, isoprenaline up to 1 µM was added. (**D**) Original recording of the inotropic effect of mephedrone and subsequently additionally applied 10 µM propranolol. Horizontal bars in (**A**–**D**) indicate time axis in minutes (min). (**E**) Force of contraction in percent of control (Ctr; pre-drug value) of the inotropic effect of mephedrone (Me) and additionally applied propranolol (Me + P). (**F**) Force of contraction in percent of control of the inotropic effect of mephedrone (Me) and mephedrone in the additional presence of cocaine (Co + Me). * *p* < 0.05. Data are shown as mean ± SD, and expressed as the percentage of control. The numbers in the bars indicate the number of experiments.

First, we wanted to know whether muscarinic receptors are involved in the negative inotropic effects of DOI or DOM, since stimulation of muscarinic receptors has been demonstrated to exert negative inotropic effects in the human atrium. Accordingly, the muscarinic receptor antagonist atropine (10 µM) was added first, followed by the addition of DOI or DOM. The original recordings are shown in Figure 5A,B. It is evident that atropine is unable to prevent the negative inotropic effects of DOI or DOM. This indicates that neither DOI nor DOM activated muscarinic receptors.

With regard to mephedrone, our initial hypothesis was that it might act in a manner analogous to that of amphetamine. Consequently, at the end of the experiment, we added propranolol, a β-adrenoceptor antagonist. This propranolol completely reversed the positive inotropic effect of mephedrone (Figure 5D,E). Therefore, it can be concluded that the increase in contractile force caused by mephedrone is mediated by β-adrenoceptors. However, as with amphetamine, the effect of mephedrone could simply be due to the release of noradrenaline from cardiac stores. This release of noradrenaline can be inhibited by cocaine. Accordingly, experiments were performed as shown in Figure 5C. First, the human atrial preparations were treated with mephedrone to confirm that mephedrone exerts a positive inotropic effect in each preparation. Subsequently, the mephedrone was washed out, cocaine was added, and mephedrone was reapplied. Now, the inotropic effect of mephedrone was greatly diminished, as evidenced by the original recording of Figure 5C and summarized in Figure 5F.

## 3. Discussion

### 3.1. Main New Findings

The present study appears to demonstrate, for the first time, that both DOI and DOM can induce negative inotropic effects in the human heart. Moreover, to the best of our knowledge, we demonstrate for the first time that mephedrone can enhance the contractile force in the human heart.

### 3.2. Mechanism

Early studies of DOI in experimental animal models demonstrated cardiovascular effects such as increased blood pressure or decreased heart rate [15,16,17]. In cats, these effects were attributed to possible central activation of the sympathetic nervous system through stimulation of central 5-HT_2_ serotonin receptors, as confirmed by the inhibitory effect of ketanserin, a 5-HT_2_ serotonin receptor antagonist [17]. In rats, the cardiovascular effects of DOI have also been proposed to be centrally mediated [15,16]. In guinea pig papillary muscles, the effect of DOI on monophasic action potentials was measured in the presence of antagonists of M-cholinoceptors and of α- and β-adrenoceptors. Under these conditions, DOI shortened the duration of action potentials [18]. Unfortunately, the contractile force was not measured in this study. However, these animal experiments indicated that DOI has an electrophysiological effect on the heart [18]. It should be noted that there are regional and species-specific differences. For instance, the aforementioned study focused on ventricular function in guinea pigs, whereas our study concentrated on atrial function in humans. It is therefore important to ascertain whether the negative inotropic effect of DOI in the human atrium is simply due to muscarinic receptor stimulation. Activation of muscarinic receptors would result in a negative inotropic effect. Consequently, atropine was tested for its potential to influence the contractile effect of DOI, but no discernible impact was observed. This is consistent with the inability of DOI to bind to muscarinic receptors [4]. Nevertheless, this experiment is considered instructive. One could argue that DOI could have released acetylcholine from cardiac stores and that this released acetylcholine would then have led to a negative inotropic effect by activating M_2_ muscarinic receptors, opening potassium channels, shortening the duration of the action potential and thereby shortening the time to reach peak tension and the time of relaxation, and finally reducing the contractile force. However, this simple signaling could be ruled out for DOI by adding atropine. For the same reason, atropine and DOM were also tested. This was of particular importance since apparently no studies on the binding of DOM to M-cholinoceptors had been published. But as with DOI, atropine had no effect on the effects of DOM.

Moreover, it is unlikely that DOI or DOM act as Ca^2+^-sensitizing drugs, as Ca^2+^-sensitizers are expected to elicit positive inotropic effects, or that they act as potassium channel inhibitors, as these types of drugs would prolong the action potential and thereby could increase cytosolic Ca^2+^, resulting in an increased time of relaxation and an enhanced contractile force. This was reported, for example, for the potassium channel inhibitor dofetilide [19,20]. Furthermore, isoprenaline was applied at a submaximal concentration to prestimulate basal cAMP levels, thereby obtaining a response that was in close proximity to the EC_50_ value. In this linear region of the concentration response curve, there is a high probability of detecting even minor cAMP alterations. The additional administration of DOI and DOM then at least showed that we had not overlooked a potential cAMP-mediated positive inotropic effect of DOI and DOM.

Given the assumption that the effects of DOI or DOM are centrally mediated, it is reasonable to conclude that DOI or DOM do not exert a positive inotropic effect in isolated human atrial preparations, as the central nervous system is no longer a controlling factor in these isolated atrial preparations. Moreover, the preparations were electrically stimulated. Consequently, the beating rate remained constant throughout the duration of the experiments. DOM can at least stimulate 5-HT_2_ receptors directly, independent of intermediates [7]. However, in the isolated, electrically stimulated human atrium, serotonin only increases the contractile force, but does not decrease it. Moreover, the positive inotropic effect of serotonin in isolated human atrial preparations is mediated by 5-HT_4_ serotonin receptors, rather than by 5-HT_2_ serotonin receptors [21].

Furthermore, it could be assumed that long-term consumption of DOI or DOM permanently stimulates the cardiac 5-HT_2B_ receptors, which could potentially lead to the proliferation of interstitial cells in the heart valves. This could subsequently result in their insufficiency and ultimately lead to heart failure (discussed in [21]). However, there have been no reported cases of this occurring in any DOI or DOM user to date.

If DOI or DOM were to activate 5-HT_2A_ receptors in the human coronary arteries, contraction of the coronary arteries and subsequent ischemia would be expected. At least with regard to DOM, a concentration-dependent vasoconstrictive effect could be demonstrated in isolated umbilical veins of sheep [22]. Therefore, it is also conceivable that DOM could cause coronary vasoconstriction, which could manifest as angina pectoris, myocardial infarction, or cardiac arrhythmias. In contrast to DOI, DOM has not been studied on a broad spectrum of G-protein-coupled receptors, which would allow a more detailed understanding of the mechanism of DOM.

Mephedrone, like its parent compounds amphetamine or cathinone [1], exerted a positive inotropic effect in human atrial preparations. As with amphetamine and cathinone [1], it is hypothesized that the positive inotropic effect of mephedrone is indirect. Mephedrone releases cardiac noradrenaline, and this noradrenaline increases the contractile force via β-adrenoceptors. This assumption is supported by the finding that propranolol inhibited the positive inotropic effect of mephedrone in human atrial preparations. It is unlikely that mephedrone acted as an agonist at adrenoceptors, since the positive inotropic effect of mephedrone was largely abolished in the additional presence of cocaine. It is believed that cocaine inhibits the monoamine transporters of the plasma membrane, thereby preventing the interaction of mephedrone with these transporter proteins. In addition, the potential uptake of mephedrone and subsequent interaction with the vesicular monoamine transporter would also be prevented by cocaine [23]. This indirect mechanism is also consistent with previous findings in rats, in which mephedrone increased both heart rate and blood pressure [24]. A release of noradrenaline would explain both the increased heart rate through activation of cardiac β-adrenoceptors and the increased blood pressure through activation of vascular α-adrenoceptors.

### 3.3. Clinical Relevance

Mephedrone can be taken orally, snorted, or injected intramuscularly [25,26] and has been associated with fatal intoxications [27]. In addition, cardiac side effects such as increased blood pressure [26,28] and tachycardia have been reported in humans [26,29]. These cardiovascular effects of mephedrone can be attributed, at least in part, to our findings that mephedrone is capable of releasing cardiac noradrenaline. The endogenously released noradrenaline can then lead to hypertension and tachycardia in humans. Clinically, it might be relevant that mephedrone can directly stimulate 5-HT_2_ serotonin receptors and indirectly (via the release of noradrenaline) α_2_-adrenoceptors. Both receptor types, when acting alone or in combination, have been observed to constrict the human coronary arteries [30]. It can be thus concluded that mephedrone may cause angina pectoris and its associated consequences directly via stimulation of the 5-HT_2_ receptor and indirectly via the α_2_-adrenoceptor.

Mephedrone is a substrate of the liver enzyme CYP2D6 [31]. It can be reasonably inferred that CYP2D6 inhibitors may contribute to an increase in the cardiac side effects of mephedrone. By around 2007, mephedrone had become widely available on the illicit drug market [25,29]. In addition, mephedrone is generally not consumed (abused) alone, but often in combination with other drugs, such as 3,4-methylenedioxymethamphetamine (MDMA) [32]. In a recent report, it was demonstrated that MDMA may also release cardiac noradrenaline. Consequently, MDMA and mephedrone may have a synergistic effect on noradrenaline release [33].

Regarding DOM, there are old but important data that DOM increases blood pressure and heart rate in humans [6]. This may be due to the central effects of DOM. Nevertheless, it can be reasonably predicted that DOM should decrease the contractile force of the human heart. This hypothesis could be tested invasively (cardiac catheterization) or non-invasively (echocardiography) for DOM and/or DOI in patients suffering from DOM or DOI intoxication. However, this has not yet been reported in the literature. One would expect to find heart failure with reduced ejection fraction in these patients.

### 3.4. Limitations of the Study

While we were able to rule out the involvement of M_2_ muscarinic receptors in the negative inotropic effects of DOI and DOM, the underlying mechanism of these effects remains unclear. Performing electrophysiological studies on human cardiomyocytes would be helpful in this case, but this was beyond the scope of the present study. Furthermore, the ventricular effects of DOI, DOM, and mephedrone in patients have not been investigated due to the unavailability of the necessary tissue samples within our hospital.

## 4. Materials and Methods

### 4.1. Contractile Studies on Human Preparations

Our methods for contraction studies on human atrial preparations have been published several times and have not been modified here [1,33,34]. Briefly, the bathing solution of the organ baths (10-mL double-wall glass tissue chambers) contained 119.8 mM NaCI, 5.4 mM KCI, 1.8 mM CaCl_2_, 1.05 mM MgCl_2_, 0.42 mM NaH_2_PO_4_, 22.6 mM NaHCO_3_, 0.05 mM Na_2_EDTA, 0.28 mM ascorbic acid, and 5.05 mM glucose. To stabilize a pH of 7.4, the solution was continuously gassed with 95% O_2_ and 5% CO_2_ and tempered to 37 °C. To record isometric contractions, the atrial preparations were attached to inductive force transducers and pre-stretched to the length of their individual maximal contractile force. Atrial preparations were electrically stimulated at 1 Hz using a bipolar stimulating electrode (5 ms rectangular pulses with a stimulation voltage of approximately 20% above threshold). Force transducer signals were recorded by a PowerLab system consisting of a bridge amplifier and a digitizer (ADInstruments, Oxford, UK). All contraction parameters were calculated using the LabChart Pro V8 software (ADInstruments, Oxford, UK). Samples were obtained from 9 male and 2 female patients, aged 62–83 years (mean age ± SD: 69.5 ± 7.1 years). Further details of patient characteristics are provided in Table 1.

This study complies with the Declaration of Helsinki and was approved by the local ethics committee (hm-bü 04.08.2005). Informed consent was obtained from all patients included in the study.

### 4.2. Data Analysis

The data shown are means ± standard deviation (SD). Statistical significance was estimated using the two-way repeated measures analysis of variance followed by Bonferroni’s multiple comparisons test. Data in Figure 5E,F were analyzed using the one-way repeated measures analysis of variance followed by Bonferroni’s multiple comparisons test. A *p*-value < 0.05 was considered significant.

### 4.3. Drugs and Materials

DOI, DOM, and mephedrone were purchased from Cayman Chemicals via LGC, Luckenwalde, Germany. All other chemicals were of the highest commercially available purity grade. Deionized water was used throughout the experiments. Stock solutions were prepared fresh daily.

## 5. Conclusions

In conclusion, we describe how DOI and DOM decreased contractile force and mephedrone increased contractile force in the isolated human atrium. Therefore, amphetamine derivatives may have opposite functional effects on the human heart, both of which are potentially harmful.

## Figures and Tables

**Table 1:** Patient characteristics.

Patient ID	Gender	Age (Years)	NYHA Class	CCS Angina Grading Scale	LVEF (%)	Cardiac Catheterization Findings
#1	m	83	III	III	60	3 vessel CHD
#2	m	70	III	III	73	3 vessel CHD
#3	m	72	III–IV	IV	30	3 vessel CHD, aortic valve stenosis
#4	m	62	III	III	55	1 vessel CHD, aortic valve stenosis
#5	m	68	III	III	50	3 vessel CHD, atrioventricular block III
#6	m	62	III	III	60	3 vessel CHD
#7	m	65	III	III	64	3 vessel CHD, AF
#8	f	64	III	II	60	aortic valve stenosis
#9	m	64	III	III	55	3 vessel CHD, NSTEMI, paroxysmal AF
#10	f	73	IV	III	60	2 vessel CHD, NSTEMI, aortic valve insufficiency, AF
#11	m	82	III–IV	III	60	3 vessel CHD, paroxysmal AF
Mean ± SD		69.5 ± 7.1			57 ± 10	

NYHA: New York Heart Association; CCS: Canadian Cardiovascular Society; LVEF: left ventricular ejection fraction; CHD, coronary heart disease; AF: atrial fibrillation; NSTEMI: non-ST-segment elevation myocardial infarction.

## Data Availability

The raw data supporting the conclusions of this article will be made available by the authors on request.

## References

[1] Neumann J., Hußler W., Hofmann B., Gergs U. (2024). Contractile Effects of Amphetamine, Pseudoephedrine, Nor-pseudoephedrine (Cathine), and Cathinone on Atrial Preparations of Mice and Humans. J. Cardiovasc. Pharmacol..

[2] Sadzot B., Baraban J.M., Glennon R.A., Lyon R.A., Leonhardt S., Jan C.R., Titeler M. (1989). Hallucinogenic drug interactions at human brain 5-HT2 receptors: Implications for treating LSD-induced hallucinogenesis. Psychopharmacology.

[3] Canal C.E., Morgan D. (2012). Head-twitch response in rodents induced by the hallucinogen 2,5-dimethoxy-4-iodoamphetamine: A comprehensive history, a re-evaluation of mechanisms, and its utility as a model. Drug Test. Anal..

[4] Ray T.S. (2010). Psychedelics and the human receptorome. PLoS ONE.

[5] Halberstadt A.L., Geyer M.A. (2011). Multiple receptors contribute to the behavioral effects of indoleamine hallucinogens. Neuropharmacology.

[6] Snyder S.H., Faillace L., Hollister L. (1968). 2,5-Dimethoxy-4-methylamphetamine: New hallucinogenic drug. Science.

[7] Sanders-Bush E., Burris K.D., Knoth K. (1988). Lysergic acid diethylamide and 2,5-dimethoxy-4-methylamphetamine are partial agonists at serotonin receptors linked to phosphoinositide hydrolysis. J. Pharmacol. Exp. Ther..

[8] Huang J., Beng T.H. (1972). The pressor action of 2,5-dimethoxy-4-methylamphetamine in rats. J. Pharm. Pharmacol..

[9] Tadepalli A.S., Friedman E., Gershon S. (1975). Cardiovascular actions of 2,5-dimethoxy-4-methylamphetamine (DOM) in the cat. Eur. J. Pharmacol..

[10] Philogene-Khalid H.L., Hicks C., Reitz A.B., Liu-Chen L.-Y., Rawls S.M. (2017). Synthetic cathinones and stereochemistry: S enantiomer of mephedrone reduces anxiety- and depressant-like effects in cocaine- or MDPV-abstinent rats. Drug Alcohol Depend..

[11] Baumann M.H., Ayestas M.A., Partilla J.S., Sink J.R., Shulgin A.T., Daley P.F., Brandt S.D., Rothman R.B., Ruoho A.E., Cozzi N.V. (2012). The designer methcathinone analogs, mephedrone and methylone, are substrates for monoamine transporters in brain tissue. Neuropsychopharmacology.

[12] Hadlock G.C., Webb K.M., McFadden L.M., Chu P.W., Ellis J.D., Allen S.C., Andrenyak D.M., Vieira-Brock P.L., German C.L., Conrad K.M. (2011). 4-Methylmethcathinone (mephedrone): Neuropharmacological effects of a designer stimulant of abuse. J. Pharmacol. Exp. Ther..

[13] Martínez-Clemente J., Escubedo E., Pubill D., Camarasa J. (2012). Interaction of mephedrone with dopamine and serotonin targets in rats. Eur. Neuropsychopharmacol..

[14] Jacob H., Gergs U., Hofmann B., Neumann J. (2024). Lysergic acid diethylamide (LSD) has anti- β-adrenergic effects in the isolated human atrium: Abstract at the 9th German Pharm-Tox Summit 2024. Naunyn Schmiedebergs Arch. Pharmacol..

[15] Chaouche-Teyara K., Fournier B., Safar M., Dabiré H. (1994). Systemic and regional haemodynamic effects of 1-(2,5-dimethoxy-4-iodo-phenyl)-2-aminopropane (DOI) and alpha-methyl-5-HT, in the anaesthetised rat. Clin. Exp. Hypertens..

[16] Dedeoğlu A., Fisher L.A. (1991). Central and peripheral injections of the 5-HT2 agonist, 1-(2,5-dimethoxy-4-iodophenyl)-2-aminopropane, modify cardiovascular function through different mechanisms. J. Pharmacol. Exp. Ther..

[17] McCall R.B., Harris L.T. (1988). 5-HT2 receptor agonists increase spontaneous sympathetic nerve discharge. Eur. J. Pharmacol..

[18] Le Grand B., Talmant J.M., Rieu J.P., Patoiseau J.F., Colpaert F.C., John G.W. (1995). Investigation of the mechanism by which ketanserin prolongs the duration of the cardiac action potential. J. Cardiovasc. Pharmacol..

[19] Falk R.H., Decara J.M. (2000). Dofetilide: A new pure class III antiarrhythmic agent. Am. Heart J..

[20] Zhang X.-P., Wu B.-W., Yang C.-H., Wang J., Niu S.-C., Zhang M.-S. (2009). Dofetilide enhances the contractility of rat ventricular myocytes via augmentation of Na^+^-Ca^2+^ exchange. Cardiovasc. Drugs Ther..

[21] Neumann J., Hofmann B., Gergs U., Shad Kaneez F. (2017). Production and function of serotonin in cardiac cells. SEROTONIN: A Chemical Messenger between All Types of Living Cells.

[22] Zhang L., Dyer D.C. (1990). Receptor mechanisms for 5-hydroxytryptamine (5-HT) in isolated ovine umbilical vein. Eur. J. Pharmacol..

[23] Docherty J.R., Alsufyani H.A. (2021). Pharmacology of Drugs Used as Stimulants. J. Clin. Pharmacol..

[24] Varner K.J., Daigle K., Weed P.F., Lewis P.B., Mahne S.E., Sankaranarayanan A., Winsauer P.J. (2013). Comparison of the behavioral and cardiovascular effects of mephedrone with other drugs of abuse in rats. Psychopharmacology.

[25] Dargan P.I., Sedefov R., Gallegos A., Wood D.M. (2011). The pharmacology and toxicology of the synthetic cathinone mephedrone (4-methylmethcathinone). Drug Test. Anal..

[26] Wood D.M., Greene S.L., Dargan P.I. (2011). Clinical pattern of toxicity associated with the novel synthetic cathinone mephedrone. Emerg. Med. J..

[27] Loi B., Corkery J.M., Claridge H., Goodair C., Chiappini S., Gimeno Clemente C., Schifano F. (2015). Deaths of individuals aged 16-24 years in the UK after using mephedrone. Hum. Psychopharmacol..

[28] Regan L., Mitchelson M., Macdonald C. (2011). Mephedrone toxicity in a Scottish emergency department. Emerg. Med. J..

[29] James D., Adams R.D., Spears R., Cooper G., Lupton D.J., Thompson J.P., Thomas S.H.L. (2011). Clinical characteristics of mephedrone toxicity reported to the U.K. National Poisons Information Service. Emerg. Med. J..

[30] Brunton L.L., Knollmann B.C. (2023). Goodman and Gilman’s The Pharmacological Basis of Therapeutics.

[31] Mayer F.P., Wimmer L., Dillon-Carter O., Partilla J.S., Burchardt N.V., Mihovilovic M.D., Baumann M.H., Sitte H.H. (2016). Phase I metabolites of mephedrone display biological activity as substrates at monoamine transporters. Br. J. Pharmacol..

[32] Mead J., Parrott A. (2020). Mephedrone and MDMA: A comparative review. Brain Res..

[33] Neumann J., Azatsian K., Höhm C., Hofmann B., Gergs U. (2023). Cardiac effects of ephedrine, norephedrine, mescaline, and 3,4-methylenedioxymethamphetamine (MDMA) in mouse and human atrial preparations. Naunyn Schmiedebergs Arch. Pharmacol..

[34] Neumann J., Hußler W., Azatsian K., Hofmann B., Gergs U. (2023). Methamphetamine increases force of contraction in isolated human atrial preparations through the release of noradrenaline. Toxicol. Lett..

